# Blood-brain barrier disruption: mechanistic links between Western diet consumption and dementia

**DOI:** 10.3389/fnagi.2014.00088

**Published:** 2014-05-09

**Authors:** Ted M. Hsu, Scott E. Kanoski

**Affiliations:** ^1^Neuroscience Graduate Program, University of Southern CaliforniaLos Angeles, CA, USA; ^2^Department of Biological Sciences, University of Southern CaliforniaLos Angeles, CA, USA

**Keywords:** obesity, Western diet, Alzheimer’s, hippocampus, cognitive impairment, blood-brain barrier

## Abstract

Both obesity and Alzheimer’s disease (AD) are major health burdens in Western societies. While commonly viewed as having separate etiologies, this review highlights data suggesting that intake of “Western diets”, diets high in saturated fatty acids (SFA) and simple carbohydrates, may pose a common environmental risk factor contributing to the development of both of these adverse pathologies. We discuss the effects of Western Diet intake on learning and memory processes that are dependent on the hippocampus, as well as the importance of this brain region in both obesity development and the onset of Alzheimer’s and other dementias. A putative mechanism is discussed that mechanistically links Western diet consumption, blood brain barrier (BBB) degradation, and subsequent hippocampal damage and dementia pathology.

## The relationship between Western Diet intake and cognitive impairment

The increasing prevalence of obesity in modern westernized societies has received the attention of a great deal of basic science and clinical research, primarily because of the clear relationship between obesity and serious negative health outcomes such as cardiovascular disease and Type II diabetes. Emerging research has shown that in addition to these negative outcomes, obesity is also potent risk factor for cognitive decline and various types of neurodegenerative dementias (Gustafson, 2008; Hassing et al., 2009; Sellbom and Gunstad, 2012). For example, strong positive correlations exist between body mass index (BMI) and Alzheimer’s Disease (AD) development across various age groups (Cohen et al., 2011; Benito-León et al., 2013; Reinert et al., 2013). Similarly, negative correlations are present between BMI and a variety of measures for general and specific cognitive domains (Sellbom and Gunstad, 2012; Reinert et al., 2013). Importantly, while these correlations are robust and have been found in a multitude of longitudinal and cross-sectional studies (see Sellbom and Gunstad, 2012 for review), it is not entirely clear whether cognitive impairment and elevated risk for developing dementia are traits that precede obesity development, or, rather, whether obesity development itself is causing the cognitive deficiencies and dementia onset. Further, given that high levels of adiposity and obesity are a result of (and are exacerbated by) excessive consumption of unhealthy dietary factors common to a Western diet (e.g., simple carbohydrates, saturated fatty acids, or SFA), the relative contributions of obesity and excessive Western diet consumption on cognitive impairment and AD onset are not fully understood. Controlled experiments using animal models have been useful towards beginning to sort out these important questions, as well as in illuminating potential underlying neurobiological mechanisms linking metabolic derangement to cognitive impairment.

The hippocampus, a brain region responsible for certain aspects of learning and memory, is particularly susceptible to damage by circulating toxins and metabolic disturbances and is considered one of the most vulnerable sites in early Alzheimer’s and other neurodegenerative disease development (Gómez-Isla et al., 1996; Fein et al., 2000; Price et al., 2001). The large pyramidal neurons within the hippocampus have particularly high metabolic demand, thus creating a unique metabolic profile that appears to makes these neurons especially sensitive to damage from a variety of environmental and biological insults. These and other neurons in the hippocampus rely heavily both on oxidative phosphorylation and mitochondria for energy, and abnormalities in either of these mechanisms will compromise hippocampal integrity (see Michaelis, 2012 for review). Dietary composition is one such environmental factor that can negatively impact hippocampal function. The putative causal relationship between Western Diet consumption and hippocampal insult (and/or dementia onset) is supported by recent evidence showing that the hippocampus is particularly susceptible to disruption by dietary factors, specifically foods with high levels of SFAs and simple sugars. Several rodent model studies have shown deficiencies in a variety of hippocampal-dependent memory processes following Western Diet intake, particularly in tasks that require learning and utilization of stimuli in the spatial environment (Molteni et al., 2002, 2004; Stranahan et al., 2008; Kanoski and Davidson, 2010). A series of studies, Kanoski, Davidson, and colleagues demonstrated that Western diet-induced deficits in hippocampal-dependent memory processes can arise before the onset of obesity, and before other mnemonic domains that do not rely on the hippocampus are negatively affected (see Kanoski and Davidson, 2011; Kanoski, 2012 for review). For example, after rodents were trained to learn the spatial location of food rewards in a radial arm maze task, exposure to a Western Diet impaired memory retention following only 72 h of *ad libitum* consumption (Kanoski and Davidson, 2010). Similar results have been found after 9 days of Western Diet exposure in rats tested with a different spatial memory paradigm (Morris Water Maze) (Murray et al., 2009). Interestingly, Kanoski and Davidson tested both spatial reference memory (hippocampal-dependent) and non-spatial reference memory (hippocampal-independent) memory retention ability following varying lengths of Western diet consumption. Their results showed that while spatial reference memory impairments arose and remained relatively stable after only 72 h consumption, nonspatial reference impairments were not robustly observed until after 30 days of Western diet consumption. This suggests that hippocampal dependent spatial memory is particularly sensitive to disruption by Western Diet intake and that these impairments can arise prior to the development of Western diet-induced metabolic derangements and increased adiposity.

The susceptibility of the hippocampus to damage by Western Diet intake has been further demonstrated in nonspatial learning and memory tasks that are hippocampal dependent. In a Pavlovian learning task known as negative occasion setting, a brief stimulus [e.g., tone (A+)] is reinforced when presented alone, but not reinforced when a different stimulus [e.g., a light (X)] precedes it (X− > A−) (Holland et al., 1999). Compared to low-fat, healthy chow fed controls, animals fed a Western diet were more impaired on negative occasion setting than in non-conditional discrimination task (A+, B−) that is not sensitive to hippocampal damage. These results suggest that even non-spatial hippocampal dependent learning and memory processes are vulnerable to disruption by Western Diet intake (Kanoski et al., 2010). Taken together, the particular vulnerability of the hippocampus to disruption (in either spatial or non-spatial mnemonic processes) is a robust phenomenon that has important implications towards understanding the neuronal and dietary factors contributing to Alzheimer’s and other neurodegenerative diseases.

## Neurobiological mechanisms underlying Western Diet induced memory impairment: blood-brain barrier disruption

The neurobiological mechanisms that underlie learning and memory impairment resulting from Western Diet consumption have been examined in several studies employing rodent models of diet-induced obesity (DIO). The neurotrophin, brain-derived neurotrophic factor (BDNF), is one marker whose levels are considered to correlate with hippocampus integrity. BDNF is expressed extensively within the hippocampus, hypothalamus, and cerebral cortex and has crucial roles in the survival, maintenance and growth of many types of neurons (Lee et al., 2002; Monteggia et al., 2004; Rossi et al., 2006). Research has shown that reductions in BDNF levels interfere with cellular mechanisms that putatively underlie hippocampal-dependent learning and memory processes, including long-term potentiation and neurogenesis (Rossi et al., 2006; Winocur et al., 2006). Several studies have shown that rats consuming diets high in SFAs and refined carbohydrates for several months have reduced levels of BDNF in the hippocampus (Molteni et al., 2002; Kanoski et al., 2007; Stranahan et al., 2008) and in the prefrontal cortex (Kanoski et al., 2007). Other experiments have also found altered dendritic morphology (Granholm et al., 2008), impaired synaptic plasticity (Stranahan et al., 2008), altered blood vessel structure (Freeman et al., 2011), and increased neuroinflammation (White et al., 2009; Pistell et al., 2010) in the hippocampus following Western Diet consumption. Further, glutamatergic signaling is altered in hippocampal neurons by Western Diet consumption, specifically via upregulation of synaptic clearance mechanisms and altered glutamate metabolism leading to NMDA receptor desensitization (Valladolid-Acebes et al., 2012) (see Freeman et al., 2011; Kanoski and Davidson, 2011 for a more detailed review of underlying neurobiological mechanisms).

While these various alterations in neuronal maintenance and communication may each independently contribute to impaired learning and memory function resulting from Western diet consumption (e.g., in an additive fashion), it is more plausible that these changes are all part of an *interrelated* and coordinated biological response that manifests in hippocampal dysfunction and degeneration. While many of these interrelated Western diet-induced neurological outcomes have been identified, the question remains of how consuming SFAs and simple sugars lead to this type of central nervous system dysfunction. In other words, while it is possible that circulating metabolites from the breakdown of ingested SFA and simple sugars are directly altering neuronal structure, plasticity, and function, it is also likely that short- and long-term Western diet consumption leads to secondary alterations in peripheral metabolism that can directly contribute to neuronal dysfunction in the hippocampus. Consistent with this notion, changes in peripheral blood glucose regulation are considered to be one of the primary mechanisms that link Western diet consumption to cognitive impairment and Alzheimer’s pathology (see Craft, 2009 for review). Here we focus on a putative mechanism that may link Western diet consumption to alterations in the peripheral circulation that can directly impact hippocampal integrity. First we discuss evidence showing that Western diet consumption impairs the blood brain barrier (BBB) integrity, and then we highlight a putative mechanism linking Western diet intake to BBB disruption.

### Western diet consumption and BBB impairment

The BBB consists of a specialized system of microvascular endothelial cells and protects the brain from toxic substances by limiting the entry of unwanted blood components to the brain, while simultaneously permitting CNS entry of nutrients and endocrine signals through active transport mechanisms and passive diffusion. The barrier systems that protect the brain include the BBB that separates the blood from brain extracellular fluid and the choroid plexus that prevents blood from entering cerebrospinal circulation (aka, blood-cerebrospinal fluid barrier, BcsfB; Zheng et al., 2003). Damage that causes increased permeability through the BBB or BcfB has been strongly linked to the development of AD (Bowman et al., 2007) and has also been shown to precede the development of clinical symptoms in both AD patients and transgenic mouse models of AD (Skoog et al., 1998; Ujiie et al., 2003). Several recent findings have implicated dietary and metabolic factors in damage to BBB and/or BcsfB. One longitudinal study correlated mid-life adiposity in women with BBB integrity 24 years later, demonstrating that overweight and obesity can potentially serve as a trigger for the onset of vascular disorders that affect BBB permeability later in life (Gustafson et al., 2007). Another study utilizing a rabbit model of AD found increased BBB permeability and accumulation of amyloid-B (Aβ, a major pathological hallmark of AD) in the hippocampus following extended exposure to a high cholesterol diet. Further support for the relationship between dietary and metabolic factors, the BBB, and AD comes from studies from Banks and colleagues where impaired active transport/passage of various neuroendocrine signals through the BBB, including the adipostat hormone leptin, and gut-derived hormone ghrelin, was observed in obese rodents that had consumed a high fat diet (Banks et al., 2004, 2008). Interestingly, both leptin and ghrelin have neuroprotective effects and promote synaptic plasticity via action at their receptors in the hippocampus (Shanley et al., 2001; Diano et al., 2006; Garza et al., 2008; Moult et al., 2009; Kanoski et al., 2013), suggesting that disruptions of BBB active transport systems for these peptides may contribute to cognitive impairment independent of BBB passive “leakiness”. Taken together, these results suggest that BBB and BcsfB damage caused by components of the Western Diet is one mechanism that might ultimately lead to hippocampal damage, cognitive decline, and dementia onset.

This hypothesis was directly examined in a study by Kanoski et al. (2010) where rats were placed on a Western diet for 90+ days and the integrity of the BBB was determined by, (1) mRNA expression of the primary proteins that comprise the “tight junctions” of the BBB and BcsfB, and by (2) blood-to-brain permeability of sodium fluorescein (NaFl), a fluorescent-tagged molecule normally excluded from brain entry with an intact BBB and BcsfB system. The rats fed the Western diet had decreased expression of tight junction proteins, particularly claudin 5 and claudin 12, within the BBB and choroid plexus. Moreover, within the hippocampus of Western diet fed rats, there was increased NaFl fluorescence compared to control groups, an effect not observed in the cortex or the striatum. This latter finding indicates that NaFl leaked into the brain, primarily into the hippocampus. Importantly, the Western diet-fed rats were also impaired in a hippocampal-dependent negative occasion setting task, suggesting that the diet-induced BBB disruption is accompanied by negative functional consequences in hippocampal-dependent memory tests. Collectively, these data suggests that Western diet consumption degrades the integrity of the BBB, and is also the case in Alzheimer’s disease, the hippocampus is a particularly vulnerable brain region to this type of insult.

Other more recent studies have also observed BBB damage following consumption of either a Western diet (Davidson et al., 2012) or a high SFA and cholesterol diet (Freeman and Granholm, 2012). In one of these reports by Davidson et al. (2012), susceptibility to BBB disruption was examined in a DIO and diet resistant (DR) model in rodents. Some rats that are fed a high fat or Western diet over-consume the diet and develop increased adiposity and obesity (DIO), whereas others are “resistant” to these metabolic effects (DR; Levin et al., 1997). Following training on a hippocampal-dependent negative occasion setting task and a non-hippocampal dependent simple discrimination, rats were placed on either a Western diet, or regular chow for 28 days. During this time, they were tested for memory retention at 7, 14, 21, and 28 days following Western diet (or control diet) access. In the group exposed to the Western diet, animals were designated as DIO or DR based on body weight and adiposity levels after 21 days of diet exposure (groups WD-DIO, WD-DR). All of the rats showed impaired negative occasion setting memory initially during the testing period (at 7 and 14 days) relative to performance at the end of training; however, the control and WD-DR rats recovered from this deficit. Interestingly, the animals in the WD-DIO group did not recover from the deficit, and demonstrated consistent memory impairment across all subsequent testing days. Following behavioral measurements, assessment of BBB permeability revealed elevated concentrations of NaFl leakage in the hippocampus of HE-DIO rats, but not HE-DR or chow group; this effect was not observed in the striatum and prefrontal cortex. Taken together, the data presented here show that there is varying sensitivity to a Western diet induced BBB disruption and hippocampal damage, with those more prone to over-consume a Western diet and become obese being more susceptible to both hippocampal BBB impairment and disruption in hippocampal function.

### Potential mechanisms linking Western Diet consumption, BBB impairment, and Alzheimer’s pathology

**Figure 1:**
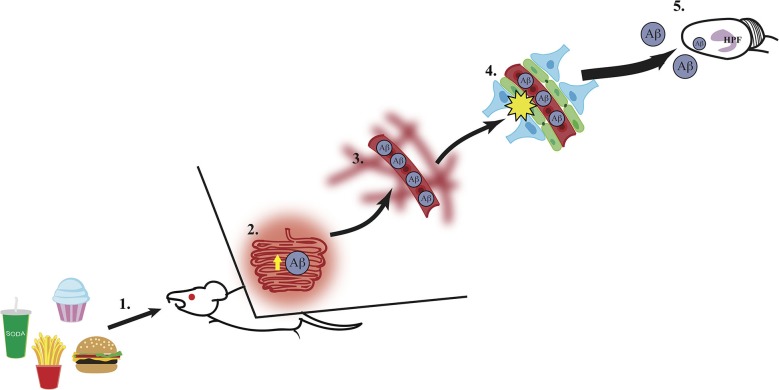
**A putative mechanism for hippocampus dysfunction by Western Diet intake. (1)** Intake of a Western Diet (simple carbohydrates, saturated fatty acids) results in **(2)** elevated secretion of amyloid-β (Aβ) from the small intestines, **(3)** thus elevating circulating Aβ levels within the vasculature system. **(4)** High circulating levels of Aβ contribute to blood-brain barrier damage via reduction of gene expression of tight junction proteins (e.g., occludin, claudin 5; illustrated in green), **(5)** which leaves the hippocampal formation (HPF) vulnerable to damage by excessive Aβ accumulation and other circulating toxins (e.g., heavy metals, inflammatory markers).

While the mechanisms linking Western diet consumption to BBB impairment, neuronal dysfunction, and memory impairment and/or dementia are not yet fully established, several pieces of evidence suggest that intake of diets high in SFAs can increase levels of circulating plasma amyloid-β (Aβ) (Takechi et al., 2008), which in turn could deteriorate the BBB and lead to hippocampal dysfunction resulting from Aβ accumulation in the hippocampus (Su et al., 1999). Research from Mamo and others has shown that rodents maintained on a high fat or Western diet had increased Aβ secretion from the small intestines absorptive epithelial cells compared to mice maintained on a low fat diet (Galloway et al., 2007, 2008). Elevated peripheral circulating levels of Aβ have been shown to disrupt the BBB in rats (Su et al., 1999). Furthermore, this disruption in the BBB can be reversed in a transgenic mouse model of AD by Aβ immunization. Additionally, Burgess and colleagues used three different transgenic mouse models of AD to determine the relationship between plasma lipid levels and Aβ in circulation. They found that while cholesterol levels were not associated with elevated levels of Aβ compared to wild-type mice, elevated very-low-density lipoprotein triglyceride levels (VLDL-TG) were found in two models for AD with abundant plasma Aβ. These transgenic mice showed excessive plasma brain amyloid deposits prior to and during elevated VLDL-TG levels (Burgess et al., 2006). These results suggest that plasma Aβ and elevated VLDL-TG levels act in concert to disrupt the BBB, allowing increased Aβ to enter the brain. Taken together, these studies show that Western diets, particularly those with high levels of SFA, can stimulate intestinal production of Aβ, increasing the level of circulating Aβ, which could increase the accumulation of Aβ within the plasma and accelerate the damage to the BBB. Given the evidence showing increased levels of circulating Aβ within the periphery during AD development, damage to the brain (particularly the hippocampus) might be caused by excessive blood to brain uptake of Aβ through a compromised BBB, again potentially caused, in part, by peripheral elevations of circulating Aβ. The finding that Aβ peptides can enter the brain of rodents from the periphery following pharmacological BBB impairment (Clifford et al., 2007) is also consistent with this possible mechanism. The direct relevance of this model to Alzheimer’s disease onset is further supported by a recent study showing that high fat diet consumption in 3xTg-AD mice increased Aβ accumulation in the hippocampus (Barron et al., 2013). Thus, these findings support a putative model (see Figure 1) in which Western diet intake increases intestinal Aβ secretion, and the subsequent elevation of peripheral circulating Aβ could potentially contribute to and accelerate BBB damage. This results in an excess of blood to brain uptake of Aβ, which in turn damages the hippocampus (a region particularly vulnerable to a leaky BBB) via increased Aβ accumulation within this region (Figure 1).

An important area for follow-up studies is to sort out the relative contributions of over consuming SFAs vs. simple sugars to BBB disruption and hippocampal impairment. Compared to a standard rodent chow “control” diet, the experimental rodents diets used in the studies described in this review contain elevated levels kcal derived from fat, ranging between 35–45% (Western diets), to 45–65% (high fat diets), with a large proportion of this fat content comprised of SFAs. However, in addition to elevated fat content, most of these diets also contain high levels of simple sugars (e.g., glucose, sucrose, fructose), whereas rodent control diets are typically composed primarily of complex carbohydrates and low percentage of kcal from fat (∼10%). A clearer picture regarding the relative contributions of these dietary factors is a crucial step forward with regards to understanding dietary effects on brain function.

## Implications of Western Diet on the development of Alzheimer’s Disease

Exponentially increasing obesity rates within Western societies over the last 30 years has been a cause for alarm (Ogden et al., 2007; Cornier et al., 2008), primarily because of associated negative health outcomes such as Type II diabetes and cardiovascular disease. Additionally, the number of people with AD is projected to increase four fold over the next 40 years, with as many as 14 million people expected to suffer from AD by 2050 (Ferri et al., 2005; Kelley and Petersen, 2007). The findings reviewed here link the onset of obesity and dementia to a common, widely prevalent phenomenon in modern Western cultures: overconsumption of foods high in SFAs and simple sugars. Given that over 2/3 of U.S. citizens today are classified as overweight or obese (Center for Disease Control and Prevention, 2012), the possibility that these individuals are also at an elevated risk for future dementia is particularly troubling. Currently, there are no effective therapies for either AD or obesity. However, research focusing on the biological links between excessive Western diet consumption, obesity, and AD development, is a promising area of focus to advance the development of effective, integrative treatments and preventative measures for both obesity and AD.

## Conflict of interest statement

The authors declare that the research was conducted in the absence of any commercial or financial relationships that could be construed as a potential conflict of interest.
